# Effects of HIV‐1 gp120 and tat on endothelial cell sensescence and senescence‐associated microRNAs

**DOI:** 10.14814/phy2.13647

**Published:** 2018-03-29

**Authors:** Jamie G. Hijmans, Kelly Stockleman, Whitney Reiakvam, Ma'ayan V. Levy, Lillian M. Brewster, Tyler D. Bammert, Jared J. Greiner, Elizabeth Connick, Christopher A. DeSouza

**Affiliations:** ^1^ Department of Integrative Physiology Integrative Vascular Biology Laboratory University of Colorado Boulder Colorado; ^2^ Department of Medicine Division of Infectious Disease University of Arizona Tucson Arizona

**Keywords:** Endothelial cells, microRNA, senescence, viral proteins

## Abstract

The aim of this study was to determine, in vitro, the effects of X4 and R5 HIV‐1 gp120 and Tat on: (1) endothelial cell senescence and (2) endothelial cell microRNA (miR) expression. Endothelial cells were treated with media without and with: R5 gp120 (100 ng/mL), X4 gp120 (100 ng/mL), or Tat (500 ng/mL) for 24 h and stained for senescence‐associated *β*‐galactosidase (SA‐*β*‐gal). Cell expression of miR‐34a, miR‐217, and miR‐146a was determined by RT‐PCR. X4 and R5 gp120 and Tat significantly increased (~100%) cellular senescence versus control. X4 gp120 significantly increased cell expression of miR‐34a (1.60 ± 0.04 fold) and miR‐217 (1.52 ± 0.18), but not miR‐146a (1.25 ± 0.32). R5 gp120 significantly increased miR‐34a (1.23 ± 0.07) and decreased miR‐146a (0.56 ± 0.07). Tat significantly increased miR‐34a (1.49 ± 0.16) and decreased miR‐146a (0.55 ± 0.23). R5 and Tat had no effect on miR‐217 (1.05 ± 0.13 and 1.06 ± 0.24; respectively). HIV‐1 gp120 (X4 and R5) and Tat promote endothelial cell senescence and dysregulation of senescence‐associated miRs.

## Introduction

Human immunodeficiency virus (HIV)‐1 infection is associated with an increased risk and prevalence of atherosclerotic cardiovascular disease (CVD) (Islam et al. 2012; Gibellini et al. 2013; Wang et al. 2015). A major factor underlying the increased CVD burden with HIV‐1‐infection is endothelial damage and dysfunction (Subramanian et al. 2012; Gibellini et al. 2013). Although the mechanisms underlying the HIV‐1‐related vasculopathy are not fully understood, HIV‐1‐associated viral proteins are known to have deleterious effects on the endothelium. Indeed, HIV‐1 gp120 and transactivator of transcription (Tat) have been shown to cause endothelial dysfunction and, in turn, have been linked to CVD pathogenesis (Kline and Sutliff 2008; Wang et al. 2015; Haser and Sumpio 2017). For example, HIV‐1 gp120 induces endothelial cell apoptosis and impairs endothelial vasodilatory capacity (Ullrich et al. 2000; Jiang et al. 2010; MacEneaney et al. 2011) and, HIV‐1 Tat promotes endothelial cell activation and enhances the development of atherosclerotic lesions (Rusnati and Presta 2002; Duan et al. 2013).

Endothelial cellular senescence is both a cause and consequence of endothelial dysfunction and atherosclerosis (Vasile et al. 2001; Minamino and Komuro 2007; Erusalimsky 2009). Senescent cells cease to perform functions necessary to maintain vascular homeostasis (Minamino and Komuro 2007). Concomitant with functional arrest, senescent endothelial cells develop a proinflammatory senescence‐associated secretory phenotype resulting in the production and release of several cytokines and proinflammatory signaling molecules, such as IL‐6 and TNF‐*α* (Prattichizzo et al. 2016; Acosta et al. 2013). These and other senescence‐associated cytokines can induce senescence in bystander endothelial cells and stimulate monocyte activation and infiltration through the vascular wall, promoting atherosclerotic lesion development (Acosta et al. 2013). Thus, endothelial cell senescence is regarded as a critical factor in the pathogenesis and progression of atherosclerotic vascular disease (Vasile et al. 2001; Minamino and Komuro 2007; Erusalimsky 2009). Accelerated senescence is a potential mechanism underlying the dysfunctional endothelial phenotype induced by HIV‐1 gp120 and Tat. However, the impact of viral proteins on endothelial cell senescence is not well understood.

MicroRNAs (miRs) are short (~22 nucleotides), endogenous, single‐stranded, noncoding RNAs that are involved in the regulation of a number of physiological and pathological processes (Kim 2005). miRs interact with mRNAs on the basis of complementary sequences between the miRNAs and the 3′‐untranslated regions (3′UTRs) of the target mRNAs resulting in downregulation of target gene expression posttranscriptionally by either mRNA degradation and/or by suppressing translation (Bartel 2004). It is now recognized that miRs, specifically miR‐34a, miR‐146a, and miR‐217, play a pivotal role in regulating endothelial cell senescence (Bhaumik et al. 2009; Menghini et al. 2009; Ito et al. 2010; Badi et al. 2015). Altered expression of these senescence‐associated miRs (SA‐miRs) has been shown to mediate endothelial senescence under various physiologic and pathologic conditions (Menghini et al. 2013). The effect of HIV‐1 viral proteins on the cellular expression of SA‐miRs, however, is currently unknown.

Accordingly, the aim of this study was to determine: (1) the effects of X4 and R5 HIV‐1 gp120 and Tat on endothelial cell senescence and (2) whether the cellular expression of SA‐miRs (miR‐34a, miR‐146a, and miR‐217) is adversely affected by these HIV‐1 viral proteins.

## Materials and Methods

### Viral Proteins

Recombinant HIV‐1 proteins Tat and Bal gp120 (R5) were obtained through the AIDS Research and Reference Reagent Program (Division of AIDS, NIAD, NIH) and gp120 Lav (X4) was purchased from Protein Sciences Corporation (Meriden, CT). To reconstitute Tat, 100 mL of PBS was bubbled with compressed N_2_ for 20 min followed by the addition of 15 mg of DTT and 100 mg of BSA and cooled on ice. Thereafter, 250 *μ*L of the PBS solution was used to dissolve the Tat. The gp120s were diluted in culture media to the desired concentrations.

### Cell culture

Human aortic endothelial cells (HAECs) were purchased from Life Technologies (ThermoFisher, Waltham, MA) and cultured in endothelial growth media (EBM‐2 BulletKit)(Lonza, Basel, Switzerland) supplemented with the 100 U/mL penicillin and 100 *μ*g/mL streptomycin under standard cell culture conditions (37°C and 5% CO_2_). Growth media were replaced 24 h after initial culture and every 2 days thereafter. Cells were serially passaged after reaching 80–90% confluence and cells were harvested for experimentation after reaching ~90% confluence on the 3rd passage. Cells were seeded into 6‐well tissue culture plates (Falcon, Corning NY) and treated with media alone or media containing HIV‐1 X4 gp120 (100 ng/mL), R5 gp120 (100 ng/mL) or Tat (500 ng/mL) for 24 h. After 24 h cells were stained with a senescence‐associated *β*‐galactacidase cytochemical stain or harvested for RNA isolation. Viral protein concentrations were similar to circulating levels in untreated HIV‐1‐seropositive adults (Oh et al. 1992).

### Senescence‐associated β‐galactacidase assay

Cellular senescence was quantified using cytochemical senescence‐associated *β*‐galactacidase (SA‐*β*‐gal) staining (Dimri et al. 1995; Debacq‐Chainiaux et al. 2009). Briefly, subconfluent cells were washed twice with 2 mL of PBS followed by a 5‐min incubation in 2 mL of 2% formaldehyde and 0.2% glutaraldehyde to fix cells. Fixed cells were washed twice with 2 mL of PBS and then incubated for 14 h with 2 mL of freshly prepared staining solution (1 mg/mL 5‐bromo‐4‐chloro‐3‐indolyl‐*β*D‐galactopyranoside in dimethylformamide, 40 mmol/L citric acid/sodium phosphate, 5 mmol/L potassium ferrocyanide, 5 mmol/L potassium ferricyanide, and 150 mmol/L NaCl, 2 mmol/L MgCl_2_) (ThermoFisher, Waltham MA). The staining solution was then removed and cells were washed twice with 2 mL of PBS and once with 1 mL of methanol and allowed to air dry. Cells were visualized by light microscopy (Zeiss, Thornwood, NY) and quantified in five random image fields for each condition. Cells with blue cytoplasmic staining were identified as senescent positive cells. Senescent cells (%) were determined as SA‐*β*‐gal positive cells divided by the total number of cells counted (Dimri et al. 1995).

### Intracellular miR‐34a, miR‐146a, and miR‐217 Expression

RNA was isolated from cells (1.0 × 10^5^) harvested from each treatment condition using the miRCURY RNA isolation kit (Exiqon, Vedbake, Denmark). Thereafter, isolated RNA concentration was determined using a Nanodrop Lite spectrophotometer (ThermoFisher, Waltham, MA) (Ye et al. 2013)**.**

Immediately after RNA isolation, 150 ng of RNA was reverse transcribed using the miScript II Reverse Transcription Kit (Qiagen, Hilden, Germany). RT‐PCR was performed using the BioRad CFX96 Touch Real Time System along with the miScript SYBR green PCR kit (Qiagen) and specific primers for miR‐34a, miR‐146a, miR‐217 (Qiagen) (Hao et al. 2014; Murphy et al. 2015; Shaker et al. 2015). All samples were assayed in duplicate. miRNA expression was quantified using the comparative Ct method and normalized to U6 (Ye et al. 2013). The fold change of each transcript was calculated as the 2^− ΔΔCt^ where fold change (AU) = 2^‐((Ct[miR experimental]‐Ct[RNU6experimental]‐ Ct[miR contol]‐Ct[RNU6control])^.

### Statistical analysis

Differences between treatments were determined by analysis of variance. Where indicated by a significant *F* value, post hoc tests with Bonferroni correction for multiple comparisons were performed. Changes in relative expression of miRs to the viral proteins were determined by two‐tailed, unpaired Student's t‐test. Data are reported as mean ± SEM for four independent HAEC experiments. Statistical significance was set a priori at *P* < 0.05.

## Results

**Figure 1:**
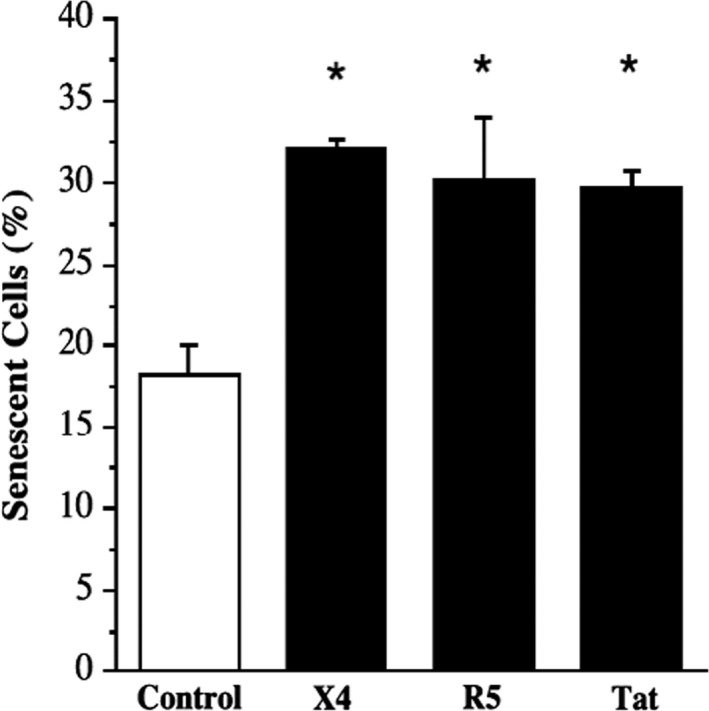
Endothelial cell senescence (%) following incubation with HIV‐1 X4 and R5 and Tat. Values are mean ± SEM (*N* = 4). *P < 0.05 versus control

Endothelial cell senescence in response to each viral protein is shown in Figure 1. The percentage of senescent cells was significantly in higher in cells treated with X4 gp120 (32 ± 1%), R5 gp120 (30 ± 3%), and Tat (30 ± 1%) proteins compared with control untreated cells (18 ± 2%). Moreover, the magnitude of increase in senescent cells was similar among the HIV‐1 viral proteins. Senescence in cells treated with denatured (boiled and sonicated) viral proteins was not different from control (data not shown).

**Figure 2:**
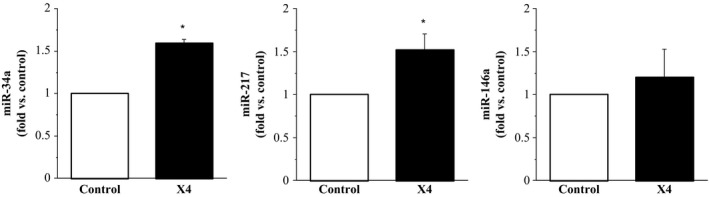
Expression of miR‐34a, miR‐217, and miR‐146a in endothelial cells treated with X4 gp120 relative to control (untreated cells). Values are mean ± SEM (*N* = 4). *P < 0.05 versus control

**Figure 3:**
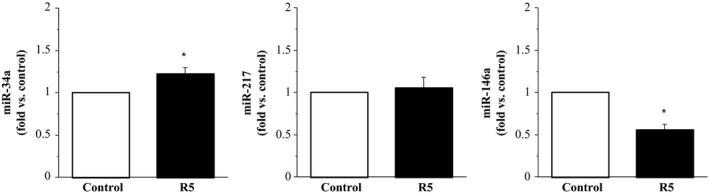
Expression of miR‐34a, miR‐217, and miR‐146a in endothelial cells treated with R5 gp120 relative to control (untreated cells). Values are mean ± SEM (*N* = 4). *P < 0.05 versus control

**Figure 4:**
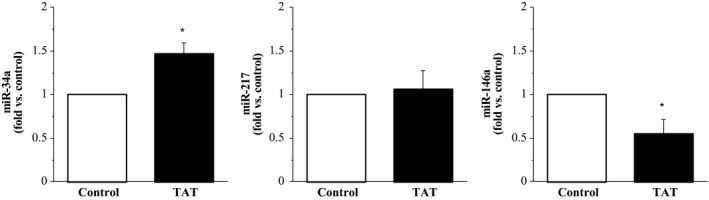
Expression of miR‐34a, miR‐217, and miR‐146a in endothelial cells treated with HIV‐1 Tat relative to control (untreated cells). Values are mean ± SEM (*N* = 4). *P < 0.05 versus control

In response to X4 gp120 treatment, cellular expression of miR‐34a (1.60 ± 0.04 fold) and miR‐217 (1.52 ± 0.18 fold) significantly increased (~60% and 50%, respectively) compared with control. There was no significant effect of X4 gp120 on the cellular expression of miR‐146a (1.25 ± 0.32 fold) (Fig. 2). In response to R5 gp120, cellular expression of miR‐34a (1.23 ± 0.07 fold) was increased (~25%; *P* < 0.05) and cellular expression of miR‐146a (0.56 ± 0.07 fold) was decreased (~80%; *P* < 0.05) compared with control. There was no significant effect of R5 gp120 on the cellular expression of miR‐217 (1.05 ± 0.13 fold) (Fig. 3). Treatment with Tat resulted in a significant increase (~50%) in the cellular expression of miR‐34a (1.49 ± 0.16 fold) and significant decrease (~80%) in the cellular expression of miR‐146a (0.55 ± 0.23 fold) compared with control. Cellular expression of miR‐217 was not significantly affected by Tat (1.06 ± 0.24 fold) (Fig. 4).

## Discussion

The primary new findings of this study are as follows: (1) HIV‐1 R5 and X4 gp120 and Tat markedly increase endothelial cell senescence; and (2) the endothelial expression signature of specific SA‐miRs is adversely altered by these proteins, thereby promoting a more senescence prone cellular phenotype. To our knowledge, this is the first study to determine the direct effects of HIV‐1 proteins on endothelial cell senescence and expression profile of SA‐miRs.

Endothelial senescence initiates and promotes a number of phenotypic changes that renders the endothelium prone to atherosclerosis (Menghini et al. 2013). For example, in addition to the release of proinflammatory cytokines, nitric oxide production has been shown to be significantly reduced in senescent endothelial cells increasing the susceptibility of the endothelium to atherosclerosis and thrombosis (Sato et al. 1993; Hoffmann et al. 2001; Matsushita et al. 2001; Krouwer et al. 2012). Furthermore, senescence negatively affects the regenerative and angiogenic capacity of the endothelium diminishing its reparative capacity and promoting atherosclerotic lesion development (Vasile et al. 2001; Minamino et al. 2002; Chang et al. 2005; Minamino and Komuro 2007; Erusalimsky 2009). Indeed, senescent endothelial cells have been found in vivo at atherosclerotic sites in both the aorta and coronary arteries (Vasile et al. 2001; Minamino et al. 2002). The results of this study demonstrate that HIV‐1 gp120 and Tat proteins accelerate endothelial cell senescence. The percentage of SA‐*β*‐gal stained HAECs markedly increased after exposure to both X4 and R5 gp120 and Tat. In fact, the degree of endothelial senescence induced (~30%) by each viral protein was almost identical, demonstrating remarkably similar, independent detrimental prosenescent effects. Unfortunately, we did not assess the combined effects of these viral proteins on endothelial senescence, a condition more representative of the *in vivo* endothelial HIV‐1 environment. It is possible that the synergistic effects of gp120 and Tat on endothelial senescence would be greater than the observed individual effects reported herein. However, future studies are needed to address this issue.

Cellular senescence is a highly conserved process that is tightly regulated by specific gene expression programs (Gorospe and Abdelmohsen 2011) and their associated miRNAs (Qin et al. 2012). In fact, aberrant expression of SA‐miRs is now regarded as a central feature of a senescent endothelial phenotype (Qin et al. 2012; Menghini et al. 2013). In this study we demonstrate, for the first time, the effects of HIV‐1 gp120 and Tat on endothelial expression of miR‐34a, miR‐217, and miR‐146a. These well‐established SA‐miRs have been shown to play a pivotal role in regulating senescence (Menghini et al. 2009; Ito et al. 2010; Vasa‐Nicotera et al. 2011). Both miR‐34a and miR‐217 promote, whereas miR‐146a quells endothelial cell senescence (Qin et al. 2012; Menghini et al. 2013). miR‐34a is highly expressed in endothelial cells and the degree of expression increases during cell senescence (Ito et al. 2010; Staszel et al. 2011; Menghini et al. 2013). miR‐34a targets and downregulates sirtuin‐1 (SIRT1), a major regulator of endothelial cell longevity and metabolic function (Potente and Dimmeler 2008; Ito et al. 2010; Zhao et al. 2010). SIRT1 is a class III histone deacetylase involved in the deacetylation of a variety of proteins, including NF‐kB and PPAR‐*γ* (Haigis and Guarente 2006). SIRT1 also exerts regulatory influence on FOXO3 and p53 (Chung et al. 2010; Ito et al. 2010). Decreased expression of SIRT1 associated with overexpression of miR‐34a triggers senescence in endothelial cells (Ito et al. 2010; Qin et al. 2012). A seminal finding of this study was that HIV‐1 X4 and R5 gp120 as well as Tat increased endothelial expression of miR‐34a. Our finding in HAECs that Tat induces endothelial senescence and increased expression of miR‐34a compliment and extend the results of Zhan et al. (2016) who demonstrated increased miR‐34a expression in senescent endothelial cells from HIV‐1 Tat transgenic mice. Similar to miR‐34a, miR‐217 also induces endothelial senescence through inhibition of SIRT1 (Menghini et al. 2009). Interestingly, however, unlike miR‐34a, miR‐217 expression was not uniformly affected by gp120 and Tat. Exposure to X4, but not R5 gp120 or Tat, caused an increase (~50%) in cellular expression of miR‐217. The mechanisms underlying the differential effects of these viral proteins on miR‐217 expression require further study.

Contrary to the prosenescent action of miR‐34a and miR‐217, miR‐146a inhibits senescence by targeting proteins in the BCL‐2 protein family (e.g., BCL‐wL and Bax) and NADPH oxidase 4. NADPH oxidase 4 is the most predominant NADPH oxidase isoform in endothelial cells and a potent mediator of oxidative stress‐related senescence (Vasa‐Nicotera et al. 2011; Rippo et al. 2014). miR‐146a also diminishes the senescence‐associated secretory phenotype via targeting of IRAK1, a key upstream activator of the NF‐*κ*B signaling pathway and the associated inflammatory cytokine (e.g., IL‐6, IL‐8) milieu (Bhaumik et al. 2009; Rippo et al. 2014). Thus, reduced expression of miR‐146a is a hallmark feature of senescent prone endothelial cells (Vasa‐Nicotera et al. 2011; Menghini et al. 2013). In this study, R5 gp120 and Tat, but not X4 gp120, significantly reduced (~80%) the expression of miR‐146a in endothelial cells. The expression profile of miR‐146a in response to the different viral proteins was also not uniform. Nevertheless, taken together, a clear and distinct prosenescent cellular miR signature emerged in response to gp120 and Tat exposure that is consistent with their common senescent effects. While R5 gp120 and Tat did not affect the expression of the prosenescent miR‐217, they each significantly reduced the expression of the antisenescent miR‐146a; whereas X4 gp120 increased miR‐217 expression, but had no effect on miR‐146a.

A limitation of this study is that we neither manipulated the levels of miR‐34a, miR‐217 or miR‐146a to counteract the viral protein effects nor did we assess the bioavailability of the miR target proteins in order to firmly establish the contribution of these SA‐miRs to the observed increase in senescence. Furthermore, mechanisms by which HIV proteins may alter miR expression remain to be explored. Endothelial cells express CXCR4 (Gupta et al. 1998) and CCR5 (Jones et al. 2011) suggesting that gp120 could exert its effects through triggering these receptors. Alternatively, gp120 may bind other cell surface molecules, be internalized and exert its effects intracellularly. It is well established that Tat binds cell surface molecules and is also readily endocytosed (Madani et al. 2011). Importantly, Tat is found in the peripheral blood of individuals with virus suppression on antiretroviral therapy (Madani et al. 2011) suggesting that it may contribute to endothelial dysfunction not only in untreated individuals, but in patients receiving therapy as well. The effects of HIV‐1 viral proteins on SA‐miR expression may represent an important mechanism underlying the proatherogenic vascular effects of gp120 and Tat (Wang et al. 2015; Zhan et al. 2016; Haser and Sumpio 2017). Future studies are needed to elucidate how these HIV‐1 proteins differentially disrupt cellular miR expression patterns and, in turn, compromise target proteins that regulate endothelial cell viability and function.

In conclusion, the results of this study demonstrate that HIV‐1 X4 and R5 gp120 and Tat induce endothelial cell senescence potentially through disruption of SA‐miRs. The prosenescent effects of gp120 and Tat on endothelial cells may contribute to the profound endothelial dysfunction and increased risk of atherosclerotic vascular disease associated with HIV‐1 infection.

## Conflicts of Interest

The authors have no conflicts of interest to disclose.

## References

[phy213647-bib-0001] Acosta, J. C. , A. Banito , T. Wuestefeld , P. Janich , J. P. Morton , P. Janich , et al. 2013 A complex secretory program orchestrated by the inflammasome controls paracrine senescence. Nat. Cell Biol. 15:978–990. 23770676 10.1038/ncb2784 PMC3732483

[phy213647-bib-0002] Badi, I. , I. Burba , C. Ruggeri , F. Zeni , M. Bertolotti , A. Scopece , et al. 2015 MicroRNA‐34a induces vascular smooth muscle cells senescence by SIRT1 downregulation and promotes the expression of age‐associated pro‐inflammatory secretory factors. J. Gerontol. A Biol. Sci. Med. Sci. 70:1304–1311. 25352462 10.1093/gerona/glu180

[phy213647-bib-0003] Bartel, D. P. 2004 MicroRNAs: genomics, biogenesis, mechanism, and function. Cell 116:281–297. 14744438 10.1016/s0092-8674(04)00045-5

[phy213647-bib-0004] Bhaumik, D. , G. K. Scott , S. Schokrpur , C. K. Patil , A. V. Orjalo , F. Rodier , et al. 2009 MicroRNAs miR‐146a/b negatively modulate the senescence‐associated inflammatory mediators IL‐6 and IL‐8. Aging 1:402–411. 20148189 10.18632/aging.100042 PMC2818025

[phy213647-bib-0005] Chang, M. W. , J. Grillari , C. Mayrhofer , K. Fortschegger , G. Allmaier , G. Marzban , et al. 2005 Comparison of early passage, senescent and hTERT immortalized endothelial cells. Exp. Cell Res. 309:121–136. 15964568 10.1016/j.yexcr.2005.05.002

[phy213647-bib-0006] Chung, S. , H. Yao , S. Caito , J. W. Hwang , G. Arunachalam , and I. Rahman . 2010 Regulation of SIRT1 in cellular functions: role of polyphenols. Arch. Biochem. Biophys. 501:79–90. 20450879 10.1016/j.abb.2010.05.003 PMC2930135

[phy213647-bib-0007] Debacq‐Chainiaux, F. , J. D. Erusalimsky , J. Campisi , and O. Toussaint . 2009 Protocols to detect senescence‐associated beta‐galactosidase (SA‐betagal) activity, a biomarker of senescent cells in culture and in vivo. Nat. Protoc. 4:1798–1806. 20010931 10.1038/nprot.2009.191

[phy213647-bib-0008] Dimri, G. , X. Lee , G. Basile , M. Acosta , G. Scott , C. Roskelley , et al. 1995 A biomarker that identifies senescent human cells in culture and in aging skin in vivo. Proc. Natl Acad. Sci. 92:9363–9367. 7568133 10.1073/pnas.92.20.9363 PMC40985

[phy213647-bib-0009] Duan, M. , H. Yao , G. Hu , X. Chen , A. K. Lund , and S. Buch . 2013 HIV Tat induces expression of ICAM‐1 in HUVECs: implications for miR‐221/‐222 in HIV‐associated cardiomyopathy. PLoS ONE 8:e60170. 23555914 10.1371/journal.pone.0060170 PMC3610892

[phy213647-bib-0010] Erusalimsky, J. D. 2009 Vascular endothelial senescence: from mechanisms to pathophysiology. J. Appl. Physiol. 106:326–332. 19036896 10.1152/japplphysiol.91353.2008 PMC2636933

[phy213647-bib-0011] Gibellini, D. , M. Borderi , A. Clo , S. Morini , A. Miserocchi , I. Bon , et al. 2013 HIV‐related mechanisms in atherosclerosis and cardiovascular diseases. J. Cardiovasc. Med. 14:780–790. 10.2459/JCM.0b013e3283619331 23656915

[phy213647-bib-0012] Gorospe, M. , and K. Abdelmohsen . 2011 MicroRegulators come of age in senescence. Trends Genet. 27:233–241. 21592610 10.1016/j.tig.2011.03.005 PMC3110060

[phy213647-bib-0013] Gupta, S. K. , P. G. Lysko , K. Pillarisetti , E. Ohlstein , and J. M. Stadel . 1998 Chemokine receptors in human endothelial cells. Functional expression of CXCR4 and its transcriptional regulation by inflammatory cytokines. J. Biol. Chem. 273:4282–4287. 9461627 10.1074/jbc.273.7.4282

[phy213647-bib-0014] Haigis, M. C. , and L. P. Guarente . 2006 Mammalian sirtuins–emerging roles in physiology, aging, and calorie restriction. Genes Dev. 20:2913–2921. 17079682 10.1101/gad.1467506

[phy213647-bib-0015] Hao, L. , X. G. Wang , J. D. Cheng , S. Z. You , S. H. Ma , X. Zhong , et al. 2014 The up‐regulation of endothelin‐1 and down‐regulation of miRNA‐125a‐5p, ‐155, and ‐199a/b‐3p in human atherosclerotic coronary artery. Cardiovasc. Pathol. 23:217–223. 24877885 10.1016/j.carpath.2014.03.009

[phy213647-bib-0016] Haser, G. C. , and B. Sumpio . 2017 Systemic and cell‐specific mechanisms of vasculopathy induced by human immunodeficiency virus and highly active antiretroviral therapy. J. Vasc. Surg. 65:849–859. 26994951 10.1016/j.jvs.2016.01.036

[phy213647-bib-0017] Hoffmann, J. , J. Haendeler , A. Aicher , L. Rossiql , M. Vasa , A. M. Zeiher , et al. 2001 Aging enhances the sensitivity of endothelial cells toward apoptotic stimuli: important role of nitric oxide. Circ. Res. 89:709–715. 11597994 10.1161/hh2001.097796

[phy213647-bib-0018] Islam, F. M. , J. Wu , J. Jansson , and D. P. Wilson . 2012 Relative risk of cardiovascular disease among people living with HIV: a systematic review and meta‐analysis. HIV Med. 13:453–468. 22413967 10.1111/j.1468-1293.2012.00996.x

[phy213647-bib-0019] Ito, T. , S. Yagi , and M. Yamakuchi . 2010 MicroRNA‐34a regulation of endothelial senescence. Biochem. Biophys. Res. Commun. 398:735–740. 20627091 10.1016/j.bbrc.2010.07.012

[phy213647-bib-0020] Jiang, J. , W. Fu , X. Wang , P. H. Lin , Q. Yao , and C. Chen . 2010 HIV gp120 induces endothelial dysfunction in tumour necrosis factor‐alpha‐activated porcine and human endothelial cells. Cardiovasc. Res. 87:366–374. 20083573 10.1093/cvr/cvq013 PMC2895538

[phy213647-bib-0021] Jones, K. L. , J. J. Maguire , and A. P. Davenport . 2011 Chemokine receptor CCR5: from AIDS to atherosclerosis. Br. J. Pharmacol. 162:1453–1469. 21133894 10.1111/j.1476-5381.2010.01147.x PMC3057285

[phy213647-bib-0022] Kim, V. N. 2005 MicroRNA biogenesis: coordinated cropping and dicing. Nat. Rev. Mol. Cell Biol. 6:376–385. 15852042 10.1038/nrm1644

[phy213647-bib-0023] Kline, E. R. , and R. L. Sutliff . 2008 The roles of HIV‐1 proteins and antiretroviral drug therapy in HIV‐1‐associated endothelial dysfunction. J. Investig. Med. 56:752–769. 10.1097/JIM.0b013e3181788d15 PMC2586126 18525451

[phy213647-bib-0024] Krouwer, V. J. , L. H. Hekking , M. Langelaar‐Makkinje , E. Regan‐Klapisz , and J. A. Post . 2012 Endothelial cell senescence is associated with disrupted cell‐cell junctions and increased monolayer permeability. Vasc. Cell. 4:12. 22929066 10.1186/2045-824X-4-12 PMC3527188

[phy213647-bib-0025] MacEneaney, O. J. , E. Connick , and C. A. DeSouza . 2011 Effects of HIV‐1 gp120 and protease inhibitors on apoptotic susceptibility of CD34 + hematopoietic progenitor cells. J. Acquir. Immune Defic. Syndr. 56:e49–e50. 21233630 10.1097/QAI.0b013e3181fb1cb3 PMC3038106

[phy213647-bib-0026] Madani, F. , S. Lindberg , U. Langel , S. Futaki , and A. Graslund . 2011 Mechanisms of cellular uptake of cell‐penetrating peptides. J. Biophys. 2011:414729. 21687343 10.1155/2011/414729 PMC3103903

[phy213647-bib-0027] Matsushita, H. , E. Chang , A. J. Glassford , J. P. Cooke , C. P. Chiu , and P. S. Tsao . 2001 eNOS activity is reduced in senescent human endothelial cells: Preservation by hTERT immortalization. Circ. Res. 89:793–798. 11679409 10.1161/hh2101.098443

[phy213647-bib-0028] Menghini, R. , V. Casagrande , M. Cardellini , E. Martelli , A. Terrinoni , F. Amati , et al. 2009 MicroRNA 217 modulates endothelial cell senescence via silent information regulator 1. Circulation 120:1524–1532. 19786632 10.1161/CIRCULATIONAHA.109.864629

[phy213647-bib-0029] Menghini, R. , V. Casagrande , and M. Federici . 2013 MicroRNAs in endothelial senescence and atherosclerosis. Cardiovasc. Transl. Res. 6:924–930. 10.1007/s12265-013-9487-7 23812745

[phy213647-bib-0030] Minamino, T. , and I. Komuro . 2007 Vascular cell senescence: contribution to atherosclerosis. Circ. Res. 100:15–26. 17204661 10.1161/01.RES.0000256837.40544.4a

[phy213647-bib-0031] Minamino, T. , H. Miyauchi , T. Yoshida , Y. Ishida , H. Yoshida , and I. Komuro . 2002 Endothelial cell senescence in human atherosclerosis: role of telomere in endothelial dysfunction. Circulation 105:1541–1544. 11927518 10.1161/01.cir.0000013836.85741.17

[phy213647-bib-0032] Murphy, M. S. , R. C. Casselman , C. Tayade , and G. N. Smith . 2015 Differential expression of plasma microRNA in preeclamptic patients at delivery and 1 year postpartum. Am. J. Obstet. Gynecol. 213:e361–e369. 10.1016/j.ajog.2015.05.013 25981845

[phy213647-bib-0033] Oh, S. K. , W. W. Cruikshank , J. Raina , C. G. Blanchard , W. H. Adler , J. Walker , et al. 1992 Identification of HIV‐1 envelope glycoprotein in the serum of AIDS and ARC patients. J. Acquir. Immune Defic. Syndr. 5:251–256. 1740750

[phy213647-bib-0034] Potente, M. , and S. Dimmeler . 2008 Emerging roles of SIRT1 in vascular endothelial homeostasis. Cell Cycle 7:2117–2122. 18641460 10.4161/cc.7.14.6267

[phy213647-bib-0035] Prattichizzo, F. , A. Giuliani , V. De Nigris , G. Pujadas , A. Ceka , L. La Sala , et al. 2016 Extracellular microRNAs and endothelial hyperglycaemic memory: a therapeutic opportunity? Diabetes Obes. Metab. 18:855–867. 27161301 10.1111/dom.12688 PMC5094499

[phy213647-bib-0036] Qin, B. , H. Yang , and B. Xiao . 2012 Role of microRNAs in endothelial inflammation and senescence. Mol. Biol. Rep. 39:4509–4518. 21952822 10.1007/s11033-011-1241-0

[phy213647-bib-0037] Rippo, M. R. , F. Olivieri , V. Monsurro , F. Prattichizzo , M. C. Albertini , and A. D. Procopio . 2014 MitomiRs in human inflamm‐aging: a hypothesis involving miR‐181a, miR‐34a and miR‐146a. Exp. Gerontol. 56:154–163. 24607549 10.1016/j.exger.2014.03.002

[phy213647-bib-0038] Rusnati, M. , and M. Presta . 2002 HIV‐1 Tat protein and endothelium: from protein/cell interaction to AIDS‐associated pathologies. Angiogenesis 5:141–151. 12831055 10.1023/a:1023892223074

[phy213647-bib-0039] Sato, I. , I. Morita , K. Kaji , M. Ikeda , M. Nagao , and S. Murota . 1993 Reduction of nitric oxide producing activity associated with in vitro aging in cultured human umbilical vein endothelial cell. Biochem. Biophys. Res. Commun. 195:1070–1076. 7690550 10.1006/bbrc.1993.2153

[phy213647-bib-0040] Shaker, O. , M. Maher , Y. Nassar , G. Morcos , and Z. Gad . 2015 Role of microRNAs ‐29b‐2, ‐155, ‐197 and ‐205 as diagnostic biomarkers in serum of breast cancer females. Gene 560:77–82. 25644077 10.1016/j.gene.2015.01.062

[phy213647-bib-0041] Staszel, T. , B. Zapala , A. Polus , A. Sadakierska‐Chudy , B. Kieć‐Wilk , E. Stępień , et al. 2011 Role of microRNAs in endothelial cell pathophysiology. Pol. Arch. Med. Wewn. 121:361–366. 21946298

[phy213647-bib-0042] Subramanian, S. , A. Tawakol , T. H. Burdo , S. Letendre , R. J. Ellis , and K. C. Williams . 2012 Arterial inflammation in patients with HIV. JAMA 308:379–386. 22820791 10.1001/jama.2012.6698 PMC3724172

[phy213647-bib-0043] Ullrich, C. K. , J. E. Groopman , and R. K. Ganju . 2000 HIV‐1 gp120‐ and gp160‐induced apoptosis in cultured endothelial cells is mediated by caspases. Blood 96:1438–1442. 10942389

[phy213647-bib-0044] Vasa‐Nicotera, M. , H. Chen , P. Tucci , A. L. Yang , G. Saintigny , R. Menghini , et al. 2011 miR‐146a is modulated in human endothelial cell with aging. Atherosclerosis 217:326–330. 21511256 10.1016/j.atherosclerosis.2011.03.034

[phy213647-bib-0045] Vasile, E. , Y. Tomita , L. F. Brown , O. Kocher , and H. F. Dvorak . 2001 Differential expression of thymosin beta‐10 by early passage and senescent vascular endothelium is modulated by VPF/VEGF: evidence for senescent endothelial cells in vivo at sites of atherosclerosis. FASEB J. 15:458–466. 11156961 10.1096/fj.00-0051com

[phy213647-bib-0046] Wang, T. , R. Yi , L. A. Green , S. Chelvanambi , M. Seimetz , and M. Clauss . 2015 Increased cardiovascular disease risk in the HIV‐positive population on ART: potential role of HIV‐Nef and Tat. Cardiovasc. Pathol. 24:279–282. 26233281 10.1016/j.carpath.2015.07.001 PMC4831910

[phy213647-bib-0047] Ye, X. , M. G. Hemida , Y. Qiu , P. J. Hanson , H. M. Zhang , and D. Yang . 2013 MiR‐126 promotes coxsackievirus replication by mediating cross‐talk of ERK1/2 and Wnt/beta‐catenin signal pathways. Cell. Mol. Life Sci. 70:4631–4644. 23811937 10.1007/s00018-013-1411-4 PMC11113642

[phy213647-bib-0048] Zhan, J. , S. Qin , L. Lu , X. Hu , J. Zhou , Y. Sun , et al. 2016 miR‐34a is a common link in both HIV‐ and antiretroviral therapy‐induced vascular aging. Aging 8:3298–3310. 27889708 10.18632/aging.101118 PMC5270669

[phy213647-bib-0049] Zhao, T. , J. Li , and A. F. Chen . 2010 MicroRNA‐34a induces endothelial progenitor cell senescence and impedes its angiogenesis via suppressing silent information regulator 1. Am. J. Physiol. Endocrinol. Metab. 299:E110–E116. 20424141 10.1152/ajpendo.00192.2010 PMC2904051

